# Simulations of cellulose translocation in the bacterial cellulose synthase suggest a regulatory mechanism for the dimeric structure of cellulose†
†Electronic supplementary information (ESI) available: Molecular simulation details including setup and parameters. Dynamics of the glucose ring twist. Bcs sequence alignment. See DOI: 10.1039/c5sc04558d


**DOI:** 10.1039/c5sc04558d

**Published:** 2016-01-29

**Authors:** Brandon C. Knott, Michael F. Crowley, Michael E. Himmel, Jochen Zimmer, Gregg T. Beckham

**Affiliations:** a National Bioenergy Center , National Renewable Energy Laboratory , 15013 Denver West Parkway , Golden CO 80401 , USA . Email: gregg.beckham@nrel.gov; b Biosciences Center , National Renewable Energy Laboratory , 15013 Denver West Parkway , Golden CO 80401 , USA; c Center for Membrane Biology , Department of Molecular Physiology and Biological Physics , University of Virginia , Charlottesville , VA 22980 , USA

## Abstract

The processive cycle of the bacterial cellulose synthase (Bcs) includes the addition of a single glucose moiety to the end of a growing cellulose chain followed by the translocation of the nascent chain across the plasma membrane. The mechanism of this translocation and its precise location within the processive cycle are not well understood. In particular, the molecular details of how a polymer (cellulose) whose basic structural unit is a dimer (cellobiose) can be constructed by adding one monomer (glucose) at a time are yet to be elucidated. Here, we have utilized molecular dynamics simulations and free energy calculations to the shed light on these questions. We find that translocation forward by one glucose unit is quite favorable energetically, giving a free energy stabilization of greater than 10 kcal mol^–1^. In addition, there is only a small barrier to translocation, implying that translocation is not rate limiting within the Bcs processive cycle (given experimental rates for cellulose synthesis *in vitro*). Perhaps most significantly, our results also indicate that steric constraints at the transmembrane tunnel entrance regulate the dimeric structure of cellulose. Namely, when a glucose molecule is added to the cellulose chain in the same orientation as the acceptor glucose, the terminal glucose freely rotates upon forward motion, thus suggesting a regulatory mechanism for the dimeric structure of cellulose. We characterize both the conserved and non-conserved enzyme–polysaccharide interactions that drive translocation, and find that 20 of the 25 residues that strongly interact with the translocating cellulose chain in the simulations are well conserved, mostly with polar or aromatic side chains. Our results also allow for a dynamical analysis of the role of the so-called ‘finger helix’ in cellulose translocation that has been observed structurally. Taken together, these findings aid in the elucidation of the translocation steps of the Bcs processive cycle and may be widely relevant to polysaccharide synthesizing or degrading enzymes that couple catalysis with chain translocation.

## Abstract 2

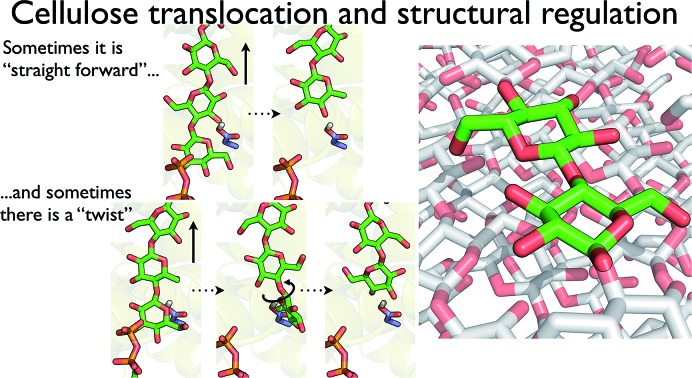
In addition to suggesting a mechanism for regulating cellulose structure, molecular simulations indicate translocation is not rate-limiting for cellulose biosynthesis.

## Introduction

Carbohydrate translocation is a ubiquitous phenomenon accomplished by myriad enzymes that synthesize, modify, and degrade polysaccharides, which generally involves threading the polymer through a tunnel or cleft. Common structural motifs are often observed even amongst enzymes of wide-ranging function, such as the lining of these tunnels and clefts with polar and aromatic residues.^1^,^2^

Cellulose is the world's most abundant polymer at a scale of several billion tons annually.^3^–^5^ In all cellulose-producing organisms, the nascent polysaccharide chain is translocated across a plasma membrane. Cellulose synthase complexes in plants generally assemble nascent cellulose chains into crystalline microfibrils. Bacteria also produce cellulose (as well as organisms from almost every kingdom of life^6^), sometimes producing higher order cellulose ‘ribbons’ from linear synthase arrays (to date, only directly observed in *Gluconacetobacter xylinus*^7^,^8^) but generally individual chains aggregate into sessile masses known as biofilms. Biofilm-embedded bacteria have important implications for human health as their tolerance to antibiotic treatments is increased.

The first crystal structure of an intact and functional cellulose synthase (Bcs) complex was published in 2013, in particular from the photosynthetic bacterium *Rhodobacter sphaeroides*.^9^ Bcs transfers a single glucose from a nucleotide-linked donor, UDP-glucose, to the end of a growing cellulose chain. This initial structure captured Bcs in an intermediate state during cellulose synthesis, as indicated by the bound cellulose chain of 18 glucose rings in length. This was thought to represent an intermediate stage of the processive cycle after glycosyl transfer and before replacing UDP with UDP-glucose. Two subsequent structures with the activator molecule cyclic-di-GMP bound to the accessory PilZ domain were consistent with a state following polymer translocation.^10^ Bcs requires the presence of cyclic-di-GMP to produce cellulose; in its absence, wild-type Bcs is almost completely inactive.^11^

**Fig. 1:**
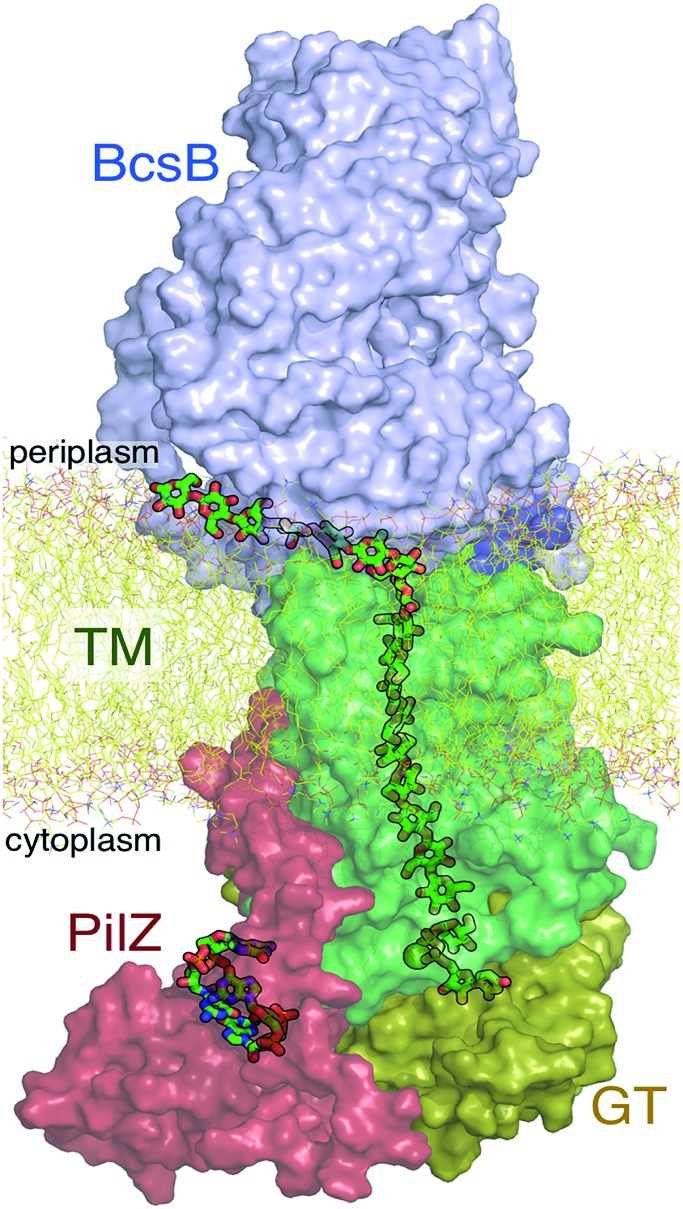
The bacterial cellulose synthase. Following addition of a single glucose ring from donor UDP-glucose at the glycosyltransferase (GT) domain, the nascent cellulose chain (shown in green ‘sticks’) is transported across the lipid bilayer (yellow ‘sticks’) through the transmembrane region (TM, green surface representation) of the BcsA domain. Also shown are the periplasmic BcsB domain and the cyclic-di-GMP (shown in green sticks) binding PilZ domain.

The A domain of Bcs (BcsA) contains eight transmembrane (TM) helices that form a narrow tunnel approximately 8 Å wide and 33 Å long that accommodates 10 glucose units of the translocating glucan (Fig. 1).^9^ Following glycosyl transfer at the enzyme active site (glycosyltransferase domain), the polysaccharide moves forward by one glucose unit.

Although the basic structural unit of cellulose is a dimer (cellobiose), the Bcs crystal structure indicates that the polymer is elongated by one glucose at a time.^9^ Historically, the dimeric nature of cellulose has led to different mechanistic and structural hypotheses, including the possibility of two active sites, formed either by one synthase or a synthase dimer.^6^ Some of these questions were answered by the crystal structures of Morgan *et al.*, namely that the synthase has only one active site and is suggestive of a mechanism wherein the donor glucose moiety binds in the same orientation every time while the acceptor orientation alternates with each round of the processive cycle.^9^ In this case, the terminal glucose would be required to rotate around its glycosidic bond after every other glycosyl transfer reaction. Corroboration of this model as well as further molecular details of the structural regulation of cellulose biosynthesis require further investigation.

**Fig. 2:**
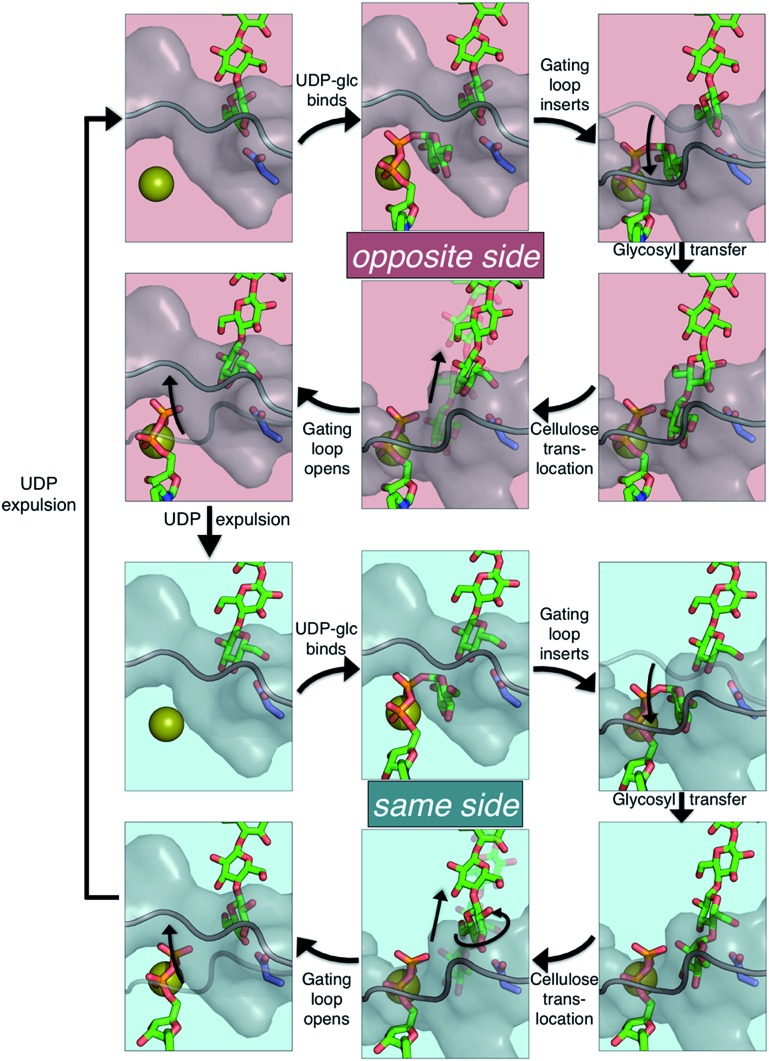
The hypothesized processive cycle of the bacterial cellulose synthase. Glycosyl transfer can add the glucose moiety from UDP-glucose in the same orientation as the acceptor glucose (‘same side’, with the hydroxymethyl of both rings on the same side) or in the opposite orientation (‘opposite side’). The following applies to both the ‘opposite side’ (top six panels) and the ‘same side’ (bottom six panels) portions of the cycle: (upper left) gating loop is open and active site is empty; (upper middle) UDP-glucose binds in the active site; (upper right) the gating loop inserts into the active site; (lower right) glycosyl transfer produces an elongated cellulose chain and UDP product; (lower middle) cellulose translocation moves the chain into the transmembrane tunnel; (lower left) gating loop opens facilitating UDP product expulsion. Alternation between the opposite and same side portions is due to Bcs adding a monomer (glucose) each round, but producing a polymer whose basic repeating unit is a dimer, cellobiose. Cellulose chain, UDP, and UDP-glucose are shown as green ‘sticks’, Mg^2+^ ion is shown as a gold sphere, Asp343 is shown in slate ‘sticks’, and the gating loop is shown as gray cartoon and surface. Note that ‘same side’ *versus* ‘opposite side’ can be most clearly differentiated in the four panels on the right side of the figure.

The available Bcs structures^9^,^10^ suggest certain details of the processive cycle by which Bcs constructs cellulose (our working hypothesis of this processive cycle is represented in Fig. 2). This cycle likely includes the opening and closing of the gating loop, a series of about eighteen residues that runs across the active site of the enzyme^10^ (shown as the solid gray ‘cartoon’ in all panels of Fig. 2). This loop has been captured structurally in three distinct positions, termed ‘open’^10^ (presumably facilitating product/reactant exchange), ‘inserted’^10^ (presumably facilitating catalysis), and ‘resting’^9^,^10^ (presumably an inactive state when cyclic di-GMP is absent). Structural evidence suggests that the transitions of the gating loop are enabled by the binding of cyclic di-GMP at the PilZ domain;^10^ the details of this regulatory mechanism are still unknown, though some evidence suggests that a salt bridge formed in the absence of c-di-GMP rigidifies the gating loop in the resting state, arresting cellulose synthesis.^10^

The mechanism of cellulose membrane translocation has been identified as a primary issue to be addressed in the field of cellulose biosynthesis.^6^ To further elucidate details of cellulose translocation in Bcs, we have performed molecular dynamics simulations and free energy calculations. We find a significant stabilization upon forward progress of the chain by one glucose unit and essentially no free energy barrier in either of the basic translocation scenarios, suggesting that translocation is not rate-limiting in the Bcs processive cycle. Our results indicate a regulatory mechanism for the dimeric structure of cellulose that is driven by steric constraints at the transmembrane tunnel entrance, thus providing an answer to a long-standing question in cellulose biosynthesis. We also characterize the roles of conserved residues that line the binding tunnel and interact with the substrate during translocation. Our results represent a step forward in the understanding of the cycle by which cellulose is synthesized biologically.

## Materials and methods

### System preparation and simulation details

All simulation systems were built and equilibrated in the molecular simulation package CHARMM.^12^ Each system was built utilizing the CHARMM-GUI^13^ Membrane Builder^14^–^16^ online tool for constructing protein/membrane complexes for molecular dynamics (MD) simulations. The major components of *R. sphaeroides* membranes are phosphatidylcholine (PC), phosphatidylglycerol (PG), and phosphatidylethanolamine (PE);^17^,^18^ past simulation work modeled this species' membrane as an equimolar mixture of POPE and POPG.^19^ For simplicity, we chose an equimolar mixture of POPE and POPC for the lipid composition in all simulations, though the results we present are not likely to be influenced by the specific chemical nature of the lipid membrane. In all cases, the approximate size of the system was 95 × 95 × 190 Å^3^ containing ∼180 000 atoms. Ions were added to produce a 0.15 M NaCl solution; the exact number of ions was slightly adjusted to achieve an overall charge-neutral system. The CHARMM-GUI^13^ also solvates the system with TIP3P water molecules.

**Fig. 3:**
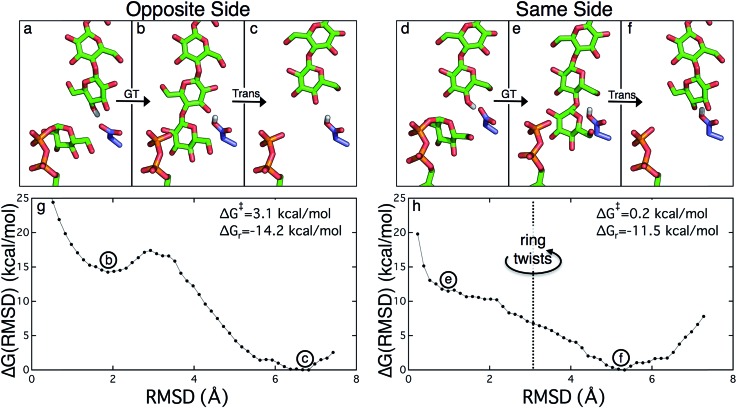
The two scenarios of glycosyl transfer (GT) and cellulose translocation (Trans) in the Bcs. The opposite side scenario is shown (a) before glycosyl transfer, (b) after glycosyl transfer, and (c) following translocation. Likewise the same side scenario is shown (d) before glycosyl transfer, (e) after glycosyl transfer, and (f) following translocation (including twist of terminal glucose ring). (g) Potential of mean force for ‘opposite side’ cellulose translocation. This PMF corresponds to translocation immediately following glycosyl transfer that produces the alternating pattern of glucose rings characteristic of native cellulose. A very modest free energy barrier precedes a large stabilization upon translocation by one glucose unit. (h) Potential of mean force for ‘same side’ cellulose translocation. This PMF corresponds to the translocation of the nascent polysaccharide immediately following glycosyl transfer resulting in the terminal glucose ring in the same configuration as the penultimate glucose (polymer in green sticks). The terminal glucose rotates upon translocation to produce the native cellulose structure. There is essentially no free energy barrier to this process and also achieves a significant stabilization.

Structural evidence suggests that the UDP-glucose donor binds in the same configuration every time, thus there are two basic scenarios of how a glucose ring can add to the cellulose chain (Fig. 2 and 3).^9^ The ‘opposite side’ configuration (as in cellulose, Fig. 3b) was constructed with the protein configuration and the cellulose chain from the crystal structure with cyclic-di-GMP and UDP bound (PDB code ; 4P00).^10^ The basis for the protein configuration in the ‘same side’ configuration (Fig. 3e) was the crystal structure with cyclic-di-GMP and UDP bound (PDB code ; 4P00).^10^ The cellulose configuration originated from the crystal structure with the cellulose chain in the ‘down’ state, pre-translocation (PDB code ; 4HG6).^9^ The two glucose rings closest to the active site were deleted, and then a single glucose ring was added in their place in the same configuration as the penultimate glucose. The system was then equilibrated for 400 ps of unrestrained MD.

After each system was built, the CHARMM-GUI^13^ minimization/relaxation protocol was followed. This consists of several rounds of minimization followed by 375 ps of MD with varying levels of harmonic restraints on different parts of the system (detailed in the ESI†).

Molecular dynamics simulations of 350 ns duration were performed utilizing the molecular simulation program NAMD^20^ for two different scenarios, both representing a glucan position following translocation. These two scenarios differ only in the orientation of the terminal glucose unit, which occupies the acceptor site in both cases. In one case the final two glucose units are in the same orientation whereas they are oppositely oriented in the other, the latter being typical of cellulose. Both of these systems were built starting with the ‘apo’ structure (lacking UDP and metal ion at the active site) with cyclic di-GMP bound (PDB code ; 4P02).^10^ The UDP and Mg^2+^ from PDB code ; 4P00 (ref. 10) were added to the active site for both systems. The ‘same side’ system was prepared by adding the terminal glucose ring from the structure without cyclic di-GMP bound (PDB code ; 4HG6,^9^ representing the state prior to translocation) and then ‘pulling’ the chain forward into the active site utilizing the ‘targeted MD’ utility from the molecular simulation package Amber12.^21^ Full details of the simulations are available in the ESI.†

### Free energy calculations

Following system-building and equilibration, we performed umbrella sampling (US) along RMSD-based coordinates using the aforementioned ‘targeted MD’ utility in Amber12.^21^ The starting configurations for each of the US windows was produced by pulling the cellulose chain backwards toward the active site targeting various RMSD values to an appropriate reference structure. For the opposite side scenario, the reference structure for the cellulose chain comes from the crystal structure with an elongated cellulose chain and lacking cyclic di-GMP (PDB code ; 4HG6).^9^ For the same side scenario, the reference structure comes from crystal structure with cyclic di-GMP and UDP bound (PDB code ; 4P00).^10^ The RMSD coordinate utilized is the C1 and C4 atoms of the glucose rings within the transmembrane region. In each case, this comprises 14 total carbon atoms that are restrained in the US simulations. Note that in each case, the restrained atoms are all in the transmembrane region; thus all atoms in the cellulose chain that are near the active site are unrestrained. Potentials of mean force (PMF) were constructed by the weighted histogram analysis method (WHAM)^22^ from the last 3 ns of 30 total ns of US.

## Results

### Free energy of cellulose translocation

The PMFs for cellulose translocation starting in the opposite and same side configurations are shown in Fig. 3, panels g and h. The opposite side case begins with the native cellulose structure (with the hydroxymethyl group alternating sides with each glucose unit); therefore the chain is pulled into the transmembrane tunnel in a straightforward fashion. Fig. 3g shows that there is only a slight 3.1 kcal mol^–1^ barrier to this process and results in a significant stabilization of 14.2 kcal mol^–1^.

The free energy profile for same side translocation (Fig. 3h) is similar and exhibits a stabilization of 11.5 kcal mol^–1^, but has no barrier to translocation. The most interesting characteristic of these simulations is that in the latter US windows (beyond an RMSD of approximately 3.0) the cellulose chain that began in the same side configuration rotates around its glycosidic bond to an opposite side configuration. This rotation was exhibited in several, but not all, of these windows. We expect that eventually this rotation would take place in all of the later windows. Thus, we have also simulated an opposite side configuration (but of opposite orientation from that shown in Fig. 3a–c), and combined that scenario's PMF from RMSD ≥3.0 with that of the same side scenario PMF from RMSD <3.0. The resulting PMF is shown in Fig. 3h.

**Fig. 4:**
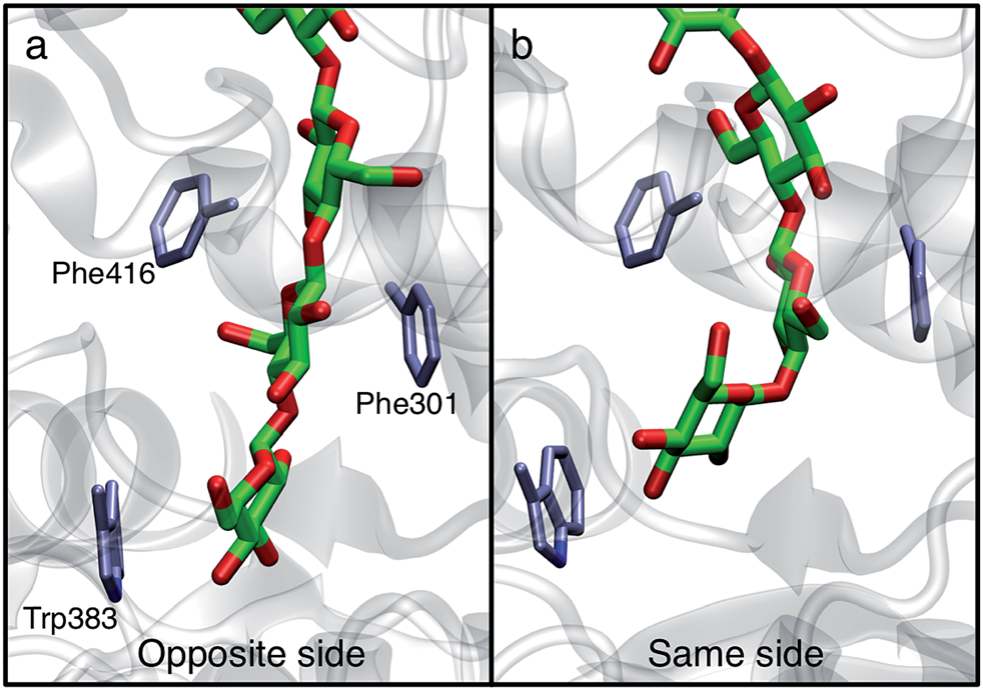
Orientation affects planarity of the cellulose chain. Snapshots from 350 ns MD simulations show how alternating each glucosyl residue's orientation allows the polysaccharide to assume a planar configuration at the transmembrane tunnel entrance.

**Fig. 5:**
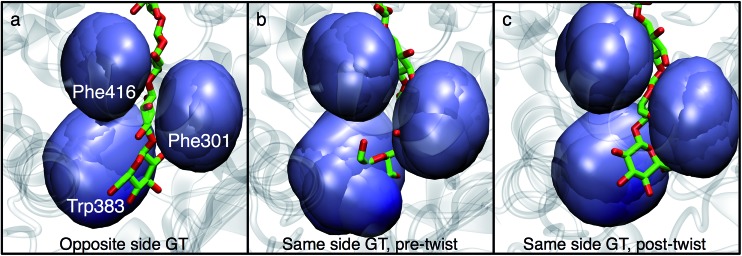
Aromatic residues at the TM tunnel entrance regulate cellulose structure. Three aromatic residues (Trp383, Phe301, and Phe416) stack on the first three residues of the nascent polysaccharide chain following translocation. The polysaccharide is shown post-translocation in three different scenarios: (a) glycosyl transfer (GT) has elongated the chain with a glucose residue in the opposite orientation as the acceptor and advanced the chain by one glucosyl unit (thus, an opposite side configuration), (b) GT has occurred in the ‘same side’ orientation; translocation has pulled the chain in but ring twisting has not yet occurred (thus, a same side configuration), and (c) same as panel b, except the terminal glucosyl residue has rotated around its acetyl linkage to produce a polysaccharide that matches the alternating orientation of glucose residues in cellulose chains (thus, an opposite side configuration). The position of each aromatic side chain is shown in ‘surface’ representation every 2 ns during the latter half of the translocation process.

In order to understand the molecular roots for the rotation of the terminal glucose seen in the US simulations, we detailed the cellulose–enzyme interactions at the TM tunnel entrance. As shown in Fig. 4, the first three glucosyl residues in the TM tunnel formed by Bcs are stabilized by carbohydrate–π stacking interactions with Trp383, Phe301, and Phe416.^1^,^6^,^23^ These three residues require the translocating polysaccharide to adopt a roughly planar configuration as it enters the TM tunnel (Fig. 5). When the glucose residues alternate orientation (as in native cellulose), planarity can be achieved (Fig. 4a), but deviations from planarity result from consecutive glucosyl residues having the same orientation (Fig. 4b). This results in not only a loss of the favorable van der Waals interactions that carbohydrate–π interactions produce, but can also produce steric clashes between the lower end of the chain with these aromatic residues (compare panels a and c with panel b in Fig. 5).

In addition to losing favorable interactions with aromatic residues at the TM tunnel entrance (primarily Trp383 on the ‘front’ side of the acceptor site), hydrogen bonds with conserved residues are also greatly impaired when the acceptor glucosyl residue is in the same orientation as its neighbor. Notable amongst these is the hydrogen bonding interaction with the backbone carbonyl oxygen of Cys318 (of the conserved FFCGS motif at the ‘back’ side of the acceptor site). As noted by Morgan *et al.*,^10^ this oxygen is positioned to hydrogen bond with the O3 hydroxyl of the acceptor site; in our 350 ns simulation in the ‘opposite side’ case, we observe this interaction as well as with the O4 hydroxyl. In the ‘same side’ orientation, these interactions are completely lost. In addition, the opposite side orientation allows the side chain oxygen of Asn298 to hydrogen bond with the hydroxymethyl of the glucosyl residue just above the acceptor site and between the side chain nitrogen with the O2 hydroxyl of the acceptor site. When in the same side orientation, all interactions between Asn298 and the acceptor site are lost. Apart from very fleeting interactions with Asp343 in the opposite orientation, neither of the 350 ns MD simulations in the post-translocation state have any significant interactions with the TED motif. This motif (residues 341–343 in Bcs) is invariant among cellulase synthase enzymes and has been noted to form hydrogen bonds with the acceptor glucose (Thr341 and Asp343) and possibly the donor (Glu342) in crystal structures of GT enzymes.^6^

### Behavior of residues lining the cellulose binding tunnel

**Fig. 6:**
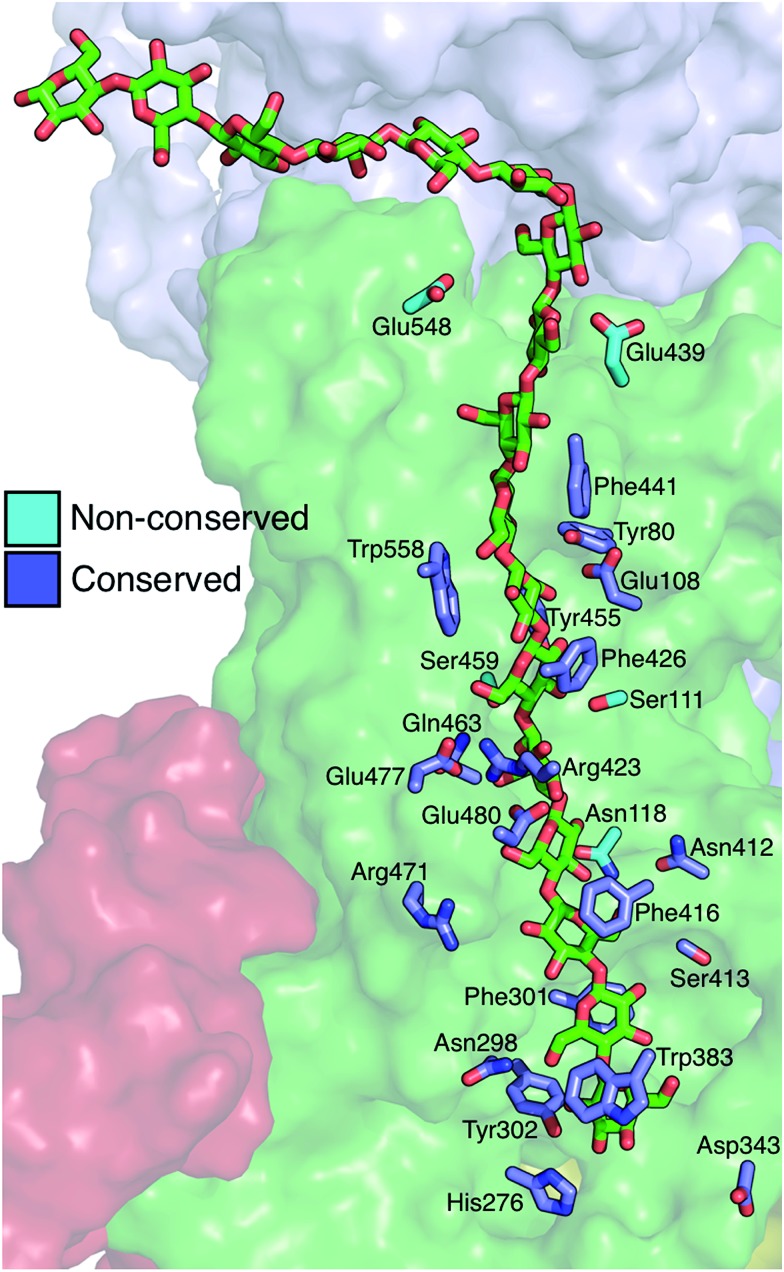
Residues directly interacting with the cellulose chain during translocation. Shown in ‘stick’ representation are the key residues that interact strongly with the cellulose chain in molecular simulations of cellulose translocation. Shown in slate are those residues that are conserved, while nonconserved residues are shown in cyan. The cellulose chain is shown in green sticks in the ‘up’ position (post-translocation).

The Bcs binding tunnel is lined with a number of aromatic and polar residues, many of which are conserved (Fig. 6, sequence aligment of Bcs can be found in the ESI†). Beginning with the acceptor site, the first three glucose rings post-translocation are stabilized by stacking interactions with Trp383 (acceptor site), Phe301, and Phe416. These three residues stack on alternate sides of the glucan. Following two glucose rings without aromatic stacking, the next three glucose rings stack with Phe426, Trp558, and Phe441, once again on alternating sides of the cellulose chain. All six of these key aromatic residues are conserved (ESI Fig. 1†). In addition, Tyr80 and Tyr455 exhibit π–π stacking while hydrogen bonding to adjacent glucosyl rings midway through the TM tunnel.

When in the active site immediately following glycosyl transfer, the most persistent interactions between the terminal glucose and the enzyme are hydrogen bonds with His276 and Cys318, as well as some limited interactions with the TED motif (341–343). Stronger interactions exist at the acceptor site (where the terminal glucose unit resides following translocation), which is formed by two conserved motifs: FFCGS (316–320) and QXXRW (379–383).^6^ In particular, carbohydrate–π stacking with Trp383 and hydrogen bonding with the backbone of Cys318 stabilize this site. In addition, Tyr302 hydrogen bonds with either the O6 or the O2 hydroxyl of the acceptor site glucose, depending on its orientation. Finally, the catalytic base Asp343 tracks with the cellulose chain end as a result of a finger helix rigid body movement, thus hydrogen bonding with the terminal sugar not only before translocation, but also during and after.

Strong hydrophilic contacts are made with the translocating polysaccharide by polar residues lining the binding tunnel. Asp343 and His276 hydrogen bond to hydroxyl groups on opposite sides of the terminal sugar ring immediately following glycosyl transfer. The two sugar rings without stacking interactions noted above (four and five glucose rings away from the active site) have a high number of hydrogen bonds with charged side chains, namely Glu477, Glu480, Arg423, and Arg471. Glu108 resides in the middle of the TM tunnel and forms persistent hydrogen bonds with the hydroxyl groups of nearby glucans. Finally, Asp548 and Glu439 reside near the periplasm side of the lipid bilayer within the TM tunnel; of the charged residues just mentioned, only the latter two are not conserved.

The polar uncharged residues that contact cellulose are generally not as well conserved; these include conserved Asn298, Ser413, and Gln463, and non-conserved Asn118, Asn412, Ser111, and Ser459. Before translocation, Asn298 ND2 forms a hydrogen bond with O2 hydroxyl one unit above the acceptor site while OD1 H bonds very intermittently with O6 hydroxyl of the acceptor site. Along the course of translocation, this residue interacts only fleetingly with the ligand and it even completely rotates away from the tunnel core at times, while post-translocation, its ND2 hydrogen bonds with the O6 hydroxyl one unit above the acceptor site. The side chain of Ser413 has some minimal hydrogen bonding with the opposite side of this same glucose ring before and during translocation, but its most persistent interactions are post-translocation, forming hydrogen bonds to the O2 hydroxyl with both its side chain hydroxyl and its backbone oxygen.

With both its backbone and side chain oxygen, Asn412 hydrogen bonds preferentially with the O6 hydroxyl of the glucose ring two units above the acceptor site, much more so than the O2/O3 hydroxyls when the sugar is in the alternate configuration. Asn118 hydrogen bonds with both the nitrogen and the oxygen of its side chain to either the O6 or the O2/O3 hydroxyl (depending on its orientation) of the glucose ring three units above the acceptor site. One glucose unit farther away from the active site, Ser111 hydrogen bonds with both its side chain and backbone oxygen to the O2/O3 hydroxyls and less so with the O6 hydroxyl. Finally, Ser459 has some fleeting hydrogen bonds with the glucose ring one unit farther away from the active site.

### The role of the finger helix

Crystal structures indicate that the movement of the finger helix facilitates the translocation of the nascent cellulose chain.^10^ The finger helix is a series of about twelve residues in the GT domain that interacts with the acceptor end of the cellulose chain. Morgan *et al.* note that in structures with the cellulose chain in the ‘up’ position (post-translocation, PDB codes ; 4P00 and ; 4P02),^10^ the finger helix is rotated such that it points towards the binding tunnel entrance. In contrast, when the cellulose chain is in the ‘down’ position (pre-translocation, PDB code ; 4HG6 (ref. 9)), the finger helix points away from the TM tunnel entrance. The difference in the position of Asp343 (at the tip of the finger helix) is approximately 5 Å, corresponding to the length of a single glucose ring.^10^

**Fig. 7:**
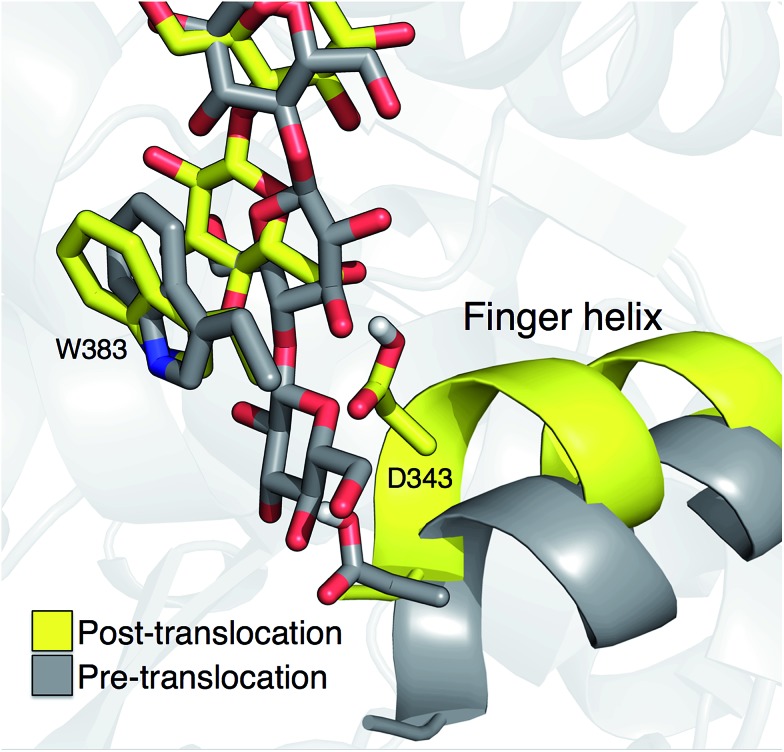
Movement of the ‘finger helix’ correlates with cellulose translocation. These images are from the opposite side translocation scenario and correspond to RMSD = 2.00 (gray) and RMSD = 6.50 (yellow), *i.e.* in the ‘down’ and ‘up’ state troughs seen in the PMF (Fig. 3g).

Our simulation results also suggest a correlation between the finger helix position and translocation progress. Shown in Fig. 7 are snapshots that correspond to the two basins of the opposite side PMF, RMSD = 2.0 (pre-translocation) and RMSD = 6.5 (post-translocation). While there is some degree of variation between the different US windows, there is a discernible trend of the finger helix moving with the cellulose chain.

## Discussion

A common feature in the PMFs for the opposite side and same side (Fig. 3) translocation scenarios is that they are both considerably downhill thermodynamically. This was also the case for TrCel7A, wherein cellulose translocation was found to be ‘driven’ forward by particularly strong interactions with polar side chains at the leading cellobiose unit that fills the so-called product sites.^24^ In the Bcs, we do not find analogous polar interactions that drive the cellulose chain forward. In the present case, however, all of the substrate binding sites of the enzyme are filled both before and after translocation, so these are not likely to provide the primary driving force(s) for translocation. Instead, the molecular roots for the propensity to translocate are likely due to some combination of two factors, both at the ends of the cellulose chain. First, translocation brings one additional glucose moiety out of the binding tunnel and into solution. Elongating the portion of the nascent chain that is exterior to the enzyme produces an increase in entropy as the polymer has an increased number of configurational states to sample. This effect is likely a small one, depending on the length of the chain; in addition, if the chain end interacts with other components in the periplasm and/or extracellular milieu,^6^ this effect would be negated. Secondly, at the opposite end of the chain, translocation brings the newly added glucose ring from the active site into the acceptor site of the binding tunnel, discussed in more detail in the following discussion.

The position of the finger helix has correlated with the translocation progress of the glucan chain in all published Bcs structures to date.^9^,^10^ Our simulations also indicate they these movements are linked. One possible source for this is the tendency of the catalytic base Asp343 to find a partner with which to share its proton. At biological pH, it is favorable for aspartic acid (p*K*_a_ < 4) to be deprotonated; following the glycosyl transfer reaction, Asp343 is protonated (it is not known how Asp343 loses this proton within the catalytic cycle, though direct transfer to the UDP product is possible). In our umbrella sampling simulations starting in the opposite side orientation, we observe intermittent, yet consistent, hydrogen bonding between Asp343 and the O6, O3, and O4 hydroxyls of the acceptor position. In so doing, Asp343 is able to delocalize some of its electron density. At various points along the translocation trajectory, Asp343 also shares this proton with water, Thr341, and Glu342.

During the course of the opposite side translocation, a persistent salt bridge between Arg499 and Glu345 suggests a possible link between gating loop motion and finger helix motion. Given the finger helix's connection to translocation as seen in crystal structures^9^,^10^ and our umbrella sampling simulations, this suggests one possible link between the catalytic activity of the Bcs and allosteric regulation by cyclic di-GMP.^10^,^11^ This salt bridge has not been observed in Bcs crystal structures to date and represents a target for future research in elucidating the molecular roots of this allostery.

The driving force for the glucose rotation seen in the same side translocation scenario are likely in the chemical nature of the entrance to the TM tunnel. It was recently noted that the acceptor position is the only binding site in BcsA in which the glucan is tightly bound, a feature that may also serve to prevent ‘backsliding’ of the chain.^6^ Conserved motifs, namely QXXRW (residues 379–383) and FFCGS (316–320), form the ‘front’ and ‘back’ sides of this binding site.^9^ As described above, when the terminal glucosyl moiety sits in the acceptor site and is oriented in the same manner as the penultimate ring, these favorable interactions are largely disrupted. In particular, the carbohydrate-aromatic stacking with Trp383 is largely lost and even results in steric hindrance. More broadly, the cellulose chain must be roughly in plane in order to be accommodated by the TM tunnel;^9^ as shown in Fig. 4 and 5, this is facilitated by alternating orientations of the glucosyl rings, thus necessitating the rotation around the acetal linkage seen in our simulations. The rotation is also likely promoted by a reduction in the internal energy of the carbohydrate chain, as previously computed for cellobiose in isolation.^25^ More details on the dynamics of the ring twist can be found in the ESI.†

In addition, simulation evidence suggests that this rotation occurs during forward motion of the newly elongated chain. We have simulated the ‘same side’ configuration in the post-translocation state (described above) for several hundred nanoseconds, and the terminal ring does not twist on this timescale, indicating that the twist is not favored to occur after translocation. Yang *et al.* utilized QM/MM calculations to estimate the free energy barrier for rotating the terminal glucosyl unit around the newly formed glycosidic bond before translocation and found this to be nearly 30 kcal mol^–1^ (nearly twice the barrier they computed for the glycosyl transfer reaction).^26^

In lipid nanodiscs (each estimated to contain one BcsA–BcsB complex), Omadjela *et al.* measured a minimal polymerization rate of ∼90 UDP molecules per s per BcsA–BcsB complex.^11^ Regardless of the precise steps and their order within the Bcs processive cycle (which may correspond more or less to Fig. 2), the rate-limiting step is likely to have a free energy barrier in the range of 14–17 kcal mol^–1^ (using transition state theory, assuming a transmission coefficient in the range of 0.01 to 1.0). Previous computational studies have estimated this barrier as 19 kcal mol^–1^,^27^ 15 kcal mol^–1^,^28^ and 16.3 kcal/mol^26^ utilizing hybrid quantum mechanics/molecular mechanics (QM/MM) calculations (the former two are for non-processive glycosyltransferases^27^,^28^ whereas the latter is for Bcs^26^). Given the exceptionally low barriers for translocation (Fig. 3), a significant outcome of the present work is that translocation is predicted to not be the rate-limiting step within in the biological production of cellulose.

At the supra-molecular level, assembly of individual cellulose chains into higher-order structures has been considered to limit the rate of cellulose biosynthesis in *Gluconacetobacter*.^29^–^31^ The genomes of many, primarily Gram-negative, bacteria contain cellulose synthase genes, including subunits required for polymer synthesis and translocation across the inner and outer membrane.^32^ Cellulose is a frequent biofilm component where it forms a 3-dimensional matrix in conjunction with curli fibers and even DNA.^33^–^35^ The organization of biofilm cellulose, *i.e.* crystalline *versus* amorphous, is currently unknown, yet it seems likely that the polymer randomly aggregates with other matrix components, rather than being ordered as observed in *Gluconacetobacter* and *Agrobacterium* species or plant cell walls. Here, the association with other biofilm components could exert a similar rate-determining effect on cellulose biosynthesis as observed during cellulose microfibril formation.

## Conclusions

Cellulose translocation in Bcs is exceptionally favorable, giving a free energy stabilization of greater than 10 kcal mol^–1^. A likely source for this is the more favorable enzyme–glucose interactions at the acceptor site *versus* the active site for the newly added glucosyl ring. In addition, when Bcs adds a glucose ring in the same configuration as the acceptor, steric constraints imposed by aromatic resides at the TM tunnel entrance produce the standard cellulose structure by rotating the terminal glucose during translocation. The linear nature of the TM tunnel necessitates the nascent polysaccharide to be roughly planar, which is achieved when each glucose ring alternates orientation. In addition, the simulation results presented here support previous structural observation regarding the correlation of the finger helix with translocation. Whether this motion actually ‘pushes’ the cellulose chain forward or is merely correlated with the chain movement is an open question for future research. Finally, comparing the free energy barriers presented here with previous calculations for the rate of cellulose synthesis *in vitro*, cellulose translocation is predicted to not be the rate-limiting step in the Bcs processive cycle.

## Supplementary Material

Supplementary information
