# Supplementary material for: Simulations of cellulose translocation in the bacterial cellulose synthase suggest a regulatory mechanism for the dimeric structure of cellulose
Source: Chem Sci. 2016 Jan 29;7(5):3108–16. doi: 10.1039/c5sc04558d (PMC4849487; doi:10.1039/c5sc04558d)
Supplement: Supplementary file 1 [file SC-007-C5SC04558D-s001.pdf]

Electronic Supplementary Material (ESI) for Chemical Science.
This journal is © The Royal Society of Chemistry 2016

Supporting Information

Simulations of cellulose translocation in the bacterial cellulose synthase suggest a regulatory mechanism for the dimeric structure of cellulose

Brandon C. Knott[1], Michael F. Crowley[2], Michael E. Himmel[2], Jochen Zimmer[3], Gregg T. Beckham[*,1]

[1]National Bioenergy Center, National Renewable Energy Laboratory, 15013 Denver West Parkway, Golden CO 80401, USA [2]Biosciences Center, National Renewable Energy Laboratory, 15013 Denver West Parkway, Golden CO 80401, USA [3]Center for Membrane Biology, Department of Molecular Physiology and Biological Physics, University of Virginia, Charlottesville, VA 22980

SIMULATION SETUP AND METHODOLOGY

All simulation systems were built and equilibrated in the molecular simulation package CHARMM.[1] Each system was built utilizing the CHARMM-GUI[2] Membrane Builder[3-5] online tool for constructing protein/membrane complexes for molecular dynamics (MD) simulations. The major components of *R. sphaeroides* membranes are phosphatidylcholine (PC), phosphatidylglycerol (PG), and phosphatidylethanolamine (PE);[6,7] past simulation work modeled this species' membrane as an equimolar mixture of POPE and POPG.[8] For simplicity, we chose an equimolar mixture of POPE and POPC for the lipid composition in all simulations, though the results we present are not likely to be influenced by the specific chemical nature of the lipid membrane. In all cases, the approximate size of the system was 95 x 95 x 190 $Å^3$ containing ~180,000 atoms. Ions were added to produce a 0.15 M NaCl solution; the exact number of ions was slightly adjusted to achieve an overall charge-neutral system. The CHARMM-GUI[2] also solvates the system with TIP3P water molecules. In all simulations, the protein, lipids, and nucleotide base (UDP) were described by the CHARMM36 with CMAP correction force field,[9-12] the carbohydrates by the CHARMM C35 force field,[13-15] and water was described with the TIP3P model.[16,17]

After each system was built, the CHARMM-GUI[2] minimization/relaxation protocol was followed. This consists of several rounds of minimization followed by 375 ps of MD with varying levels of harmonic restraints on different parts of the system. More specifically, a modified version of the CHARMM-GUI equilibration scheme was employed, in which six cycles of equilibration were performed, gradually reducing the force constants of restraints placed on various parts of the system. Cycle 1 also includes five rounds of minimization; each round of minimization includes 250 steps each of steepest descent (SD) and Adopted Basis Newton-Raphson (ABNR) minimization before MD commences. Table S1 details the timestep, number of steps, and various restraining forces on the system during minimization and equilibration.

Table S1. Simulation details for minimization and equilibration, based on CHARMM-GUI protocol.

	cycle 1	*cycle 2*	*cycle 3*	*cycle 4*	*cycle 5*	*cycle 6*
Timestep (fs)	1	1	1	2	2	2
Number of steps	25,000	25,000	25,000	50,000	50,000	50,000
FORCES (kcal/mol)						
Backbone	10.0	10.0	2.5	1.0	0.5	0.0
Sidechain	5.0	5.0	1.0	0.0	0.0	0.0
Ligand	10.0	10.0	1.0	0.0	0.0	0.0
Force (Water)	2.5	2.5	1.0	0.5	0.1	0.0
Force (Lipid tail)	2.5	2.5	1.0	0.5	0.1	0.0
Force (Lipid head)	2.5	2.5	1.0	0.5	0.1	0.0

Force (Water) is the force constant on water molecules to keep them from entering the hydrophobic core of the lipid bilayer during minimization and equilibration, F*orce (Lipid tail)* is the force constant to keep the lipid tail away from the hydrophobic core during equilibration, and *Force (Lipid head)* is the force constant to keep the lipid head groups close to their target values. Note that all of these restraints are 'turned off' for the last round of equilibration as well as all subsequent production runs.

The following parameters were utilized for the MD simulations utilizing the NAMD[18] simulation package: periodic boundary conditions, 2 fs timestep, CHARMM forcefield as described above, 12 Å nonbonded interaction cutoff, SHAKE[19,20]-constrained hydrogen bonds, 12 Å nonbonded interaction cutoff, and temperature of 300 K. The ensemble simulated is the $Np_z\Upsilon_{xy}T$ ensemble; this involves a constant number of atoms, constant pressure in the z dimension (normal to the lipid bilayer surface), constant surface tension in the plane of the bilayer, and constant temperature. The normal pressure (p_z) is set to 1 bar (controlled by the Langevin piston Nose-Hoover method), and the surface

tension in the xy plane (Υ_{xy}) is set to 0.0 dynes/cm. The three orthogonal box dimensions of the periodic cell are allowed to fluctuate independently. Long range electrostatics beyond the real-space nonbonded interaction cutoff are computed using particle mesh Ewald[21-23] summation with a 6th order B-spline interpolation and a grid size of 96x96x180.

The CHAMBER[24] program was used to convert the CHARMM protein structure file, coordinate file, and associated force field files to an AMBER topology file and coordinate file. The Sander program of the AMBER[25] software package (version 12) was used to perform the umbrella sampling (US) simulations. Amber12's[25] 'targeted MD' utility enables US along RMSD-based coordinates. The starting configurations for each of the US windows was produced by pulling the cellulose chain 'backwards' (from the 'up' state, i.e. post-translocation state) toward the active site targeting various RMSD values to the appropriate reference structure. This was done in approximately thirty increments of 0.25 Å in magnitude. Each increment was run for 10 ps. The final configuration from each of these increments is the starting point for an US simulation in that window.

The 'opposite side' configuration (as in cellulose, see main text Figure 3b) was constructed with the protein configuration and the cellulose chain from the crystal structure with cyclic-di-GMP and UDP bound (PDB code 4P00).[26] The basis for the protein configuration in the 'same side' configuration (see main text Figure 3e) was the crystal structure with cyclic-di-GMP and UDP bound (PDB code 4P00).[26] The cellulose configuration originated from the crystal structure with the cellulose chain in the 'down' state, pre-translocation (PDB code 4HG6).[27] The two glucose rings closest to the active site were deleted, and then a single glucose ring was added in their place in the same configuration as the penultimate glucose. The system was then equilibrated for 400 ps of unrestrained MD.

For the opposite side scenario, the *reference structure* for the cellulose chain comes from the crystal structure with an elongated cellulose chain and lacking cyclic di-GMP (PDB code 4HG6).[27] For the same side scenario, the *reference structure* comes from crystal structure with cyclic di-GMP and UDP bound (PDB code 4P00).[26] The RMSD coordinate utilized is the C1 and C4 atoms of the glucose rings within the transmembrane region. In each case, this comprises 14 total carbon atoms that are restrained in the US simulations. Note that in each case, the restrained atoms are all in the transmembrane region; thus all atoms in the cellulose chain that are near the active site are unrestrained. The tMD force constant is 70 kcal/mol/$Å^2$. Approximately 36 US windows were utilized for each scenario, spaced 0.25 Å apart. Potentials of mean force (PMF) were constructed by the weighted histogram analysis method (WHAM)[28] (code from Alan Grossfield[29]) from the last 3 ns of 30 total ns of US. As with the unrestrained MD simulations, the US is performed in the $Np_z\Upsilon_{xy}T$ ensemble with p_z set to 1 bar, Υ_{xy} set to 0.0 dynes/cm, and thermostat temperature of 300 K.

DYNAMICS OF THE GLUCOSE RING TWIST

French and coworkers have published extensively on energy surfaces for cellobiose.[30-33] In general, the energetics for cellobiose to twist around its glycosidic bond are tracked via ϕ-ψ two-dimensional contour maps, where ϕ is the dihedral angle H1-C1-O1-C4' and ψ is the dihedral angle H4'-C4'-O1-C1.[32] We have tracked the ϕ and ψ dihedral angles for the RMSD=3.00 Å umbrella sampling window. This is the first window wherein the rotation around the glycosidic bond is observed and thus represents the most likely point for the rotation in the physical system. We have then imposed the results on the ϕ-ψ map from French *et al.* (Figure 4 in that publication).[32] The result is seen in the figure below. The black circles represent our data for the aforementioned umbrella sampling window. The data cluster into three groups: the first is shown surrounded by blue shading, followed by the green, and finally the orange (progression shown by the red arrows). These results correspond quite nicely with the results of French *et al.* The final, low-energy, opposite-side configuration (orange shading) rests in the global minimum in the energy surface. The initial same-side state (blue shading) sits about 4-6 kcal/mol higher.

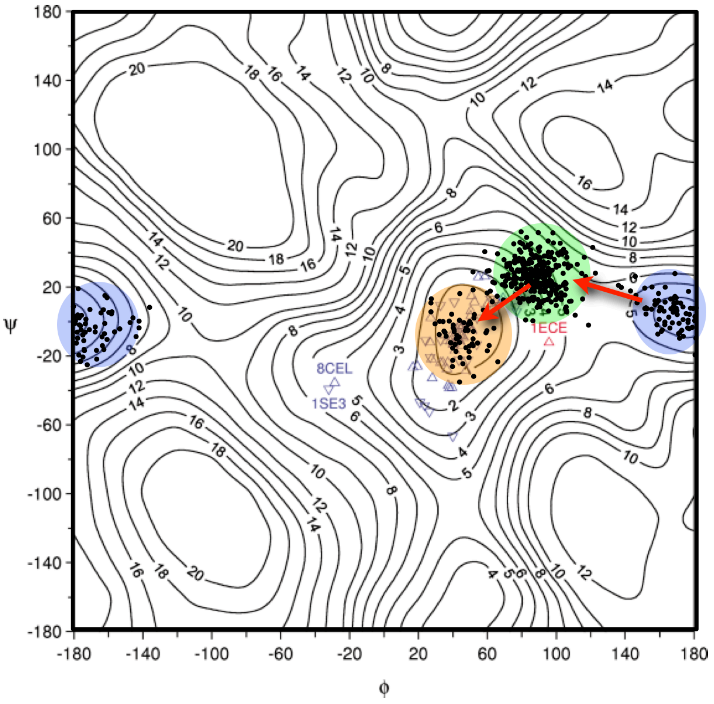

In order to analyze the dynamics of ring puckering, we have tracked the Cremer-Pople parameters for the newly added glycosyl ring during the ring twist. The results are presented below in the form of a Stoddart diagram. The data shown are for the RMSD = 3.00 Å umbrella sampling window, as in the above graph (i.e. during a complete ring twisting trajectory). As shown by this plot, there is no significant change in the ring pucker away from the $^4C_1$ conformation during the rotation, nor did we observe any non-chair conformations in other windows.

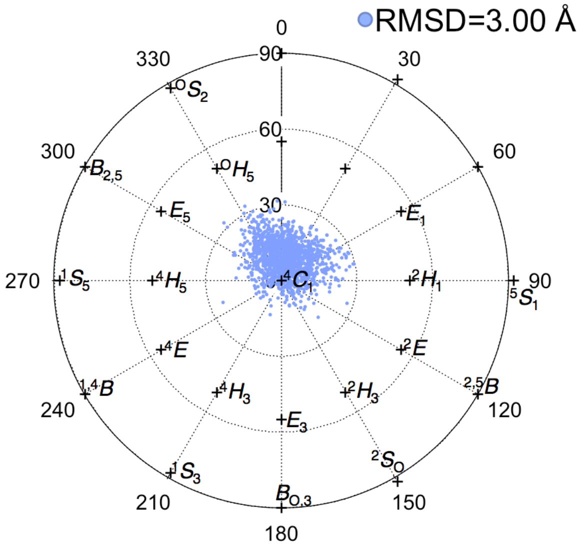

BCS SEQUENCE ALIGNMENT

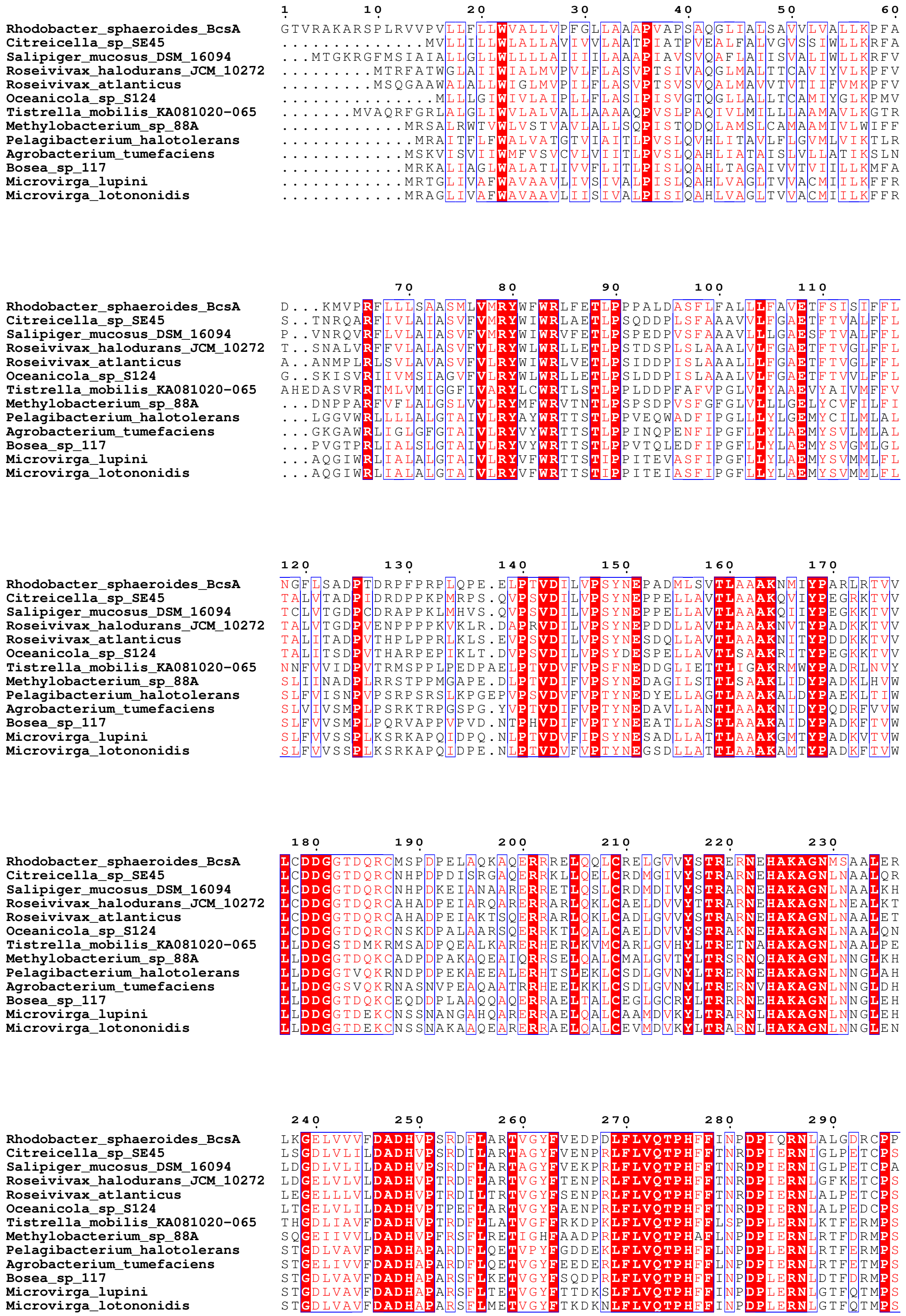

Figure S1. Sequence aligment of the bacterial cellulose synthase with the twelve closest non-redundant BLAST hits. All are from bacteria. Strictly conserved residues are shown in red block, and chemically similar residues in red text. The blue boxes indicate chemical similarity across a grouping of residues. The figure was generated with ESPript (http://espript.ibcp.fr).[34] (The sequence alignment continues on the next two pages.)

```
                                 300       310       320       330       340       350
Rhodobacter_sphaeroides_BcsA     ENEMFYGKIHRGLDRWGGAFFCGSAAVLRRRALDEAGGFAGETITEDAETALEIHSRGWK
Citreicella_sp_SE45              ENEMFYSTIHRGLDRLGGAFFCGSAALLRRKALDEVGGISGVTITEDAETALDIHSRGWE
Salipiger_mucosus_DSM_16094      ENEMFYTTIHRGLDRLGGTFFCGSAALLRRAALDEVGGISGVTITEDAETALDIHSRGWE
Roseivivax_halodurans_JCM_10272  ENEMFYGQIHRGLDRLGGAFFCGSAALLRRSALDEVGGISGETITEDAETALDIHARGWE
Roseivivax_atlanticus            ENEMFYSEIHRGLDRLGGAFFCGSAAILRRRALDEVGGIAGDTITEDAETALEIHSRGWE
Oceanicola_sp_S124               ENEMFYSEIHRGLDRMGGAFFCGSAALLRRRALDEVGGISGETITEDAETALDIHSRGWE
Tistrella_mobilis_KA081020-065   ENEMFYGMIQRGLDRWNGAFFCGSAALLRRACLEEVGGFSGLSITEDAETALDLHARGYN
Methylobacterium_sp_88A          ENEMFYSVTQRGLDKWNGSFFCGSAALLRRRALDEAGGFSGVTITEDCETAFELHSRGWN
Pelagibacterium_halotolerans     ENEMFYSILQRGLDSWNASFFCGSAALLRREALDIANGFSGRSITEDCETALDLHSKKWN
Agrobacterium_tumefaciens        ENEMFYGIIQRGLDKWNGAFFCGSAAVLRRQALKETDGFSGVSITEDCETALALHSRGWN
Bosea_sp_117                     ENEMFYGIIQRGLDKWDAAFFCGSAAVLRREALMETKGFSGISITEDCETALELHGRGWH
Microvirga_lupini                ENEMFYGVIQRGLDKWDAAFFCGSAAVLRREALQETNGFSGVSITEDCETALDLHSRGWS
Microvirga_lotononidis           ENEMFYGVIQRGLDKWDAAFFCGSAAVLRREALQETNGFSGVSITEDCETALELHSRGWT
```

```
                                 360       370       380       390       400       410
Rhodobacter_sphaeroides_BcsA     SLYIDRAMIAGLQPETFASFIQQRGRWATGMMQMLLLKNPLFRRGLGIAQRLCYLNSMSF
Citreicella_sp_SE45              SMYLNRAMIAGLQPETFASFIQQRGRWATGMMQILILKNPLFRSGLSLAQRFCYLNSMSY
Salipiger_mucosus_DSM_16094      SLYLNRAMSAGLQPETFASFIQQRGRWATGMMQILLLKNPLFRSGLSLTQRLCYLNSMSY
Roseivivax_halodurans_JCM_10272  SMYLDYAMIAGLQPETFASFIQQRGRWATGMIQMLILKNPVFRSGLTIGKRLCYLNSMSF
Roseivivax_atlanticus            SIYIERAMVAGLQPETFASFIQQRGRWATGMIQMLILKNPILRSGLSLTQRLCYLNSMSF
Oceanicola_sp_S124               SMYLDHAMIAGLQPETFASFIQQRGRWATGMIQMLILKNPMFRSGLSLTQRLCYLNSMSF
Tistrella_mobilis_KA081020-065   SVYYGKPLIAGLQPESYAAFIGQRSRWAQGMTQIFMMKNPLIKRGLRLPQRLCYLASSMF
Methylobacterium_sp_88A          SIYVDTPLIAGLQPDTLVAFIGQRSRWCQGMFQILMLKNPLFKRGLHPIQKLAYLSSMTF
Pelagibacterium_halotolerans     SIYIDRPLIAGLQPATFSSFIGQRTRWAQGMTQIMLFNFPLFKRGLSMAQRLCYMSSMMF
Agrobacterium_tumefaciens        SLYVDKPLIAGLQPATFASFIGQRSRWAQGMMQILIFRQPLFRRGLSFTQRLCYMSSTLF
Bosea_sp_117                     SVYVDKPLIAGLQPETFASFIGQRSRWAQGMYQILRFRFPPGKRGLSIPQRLCYMSSTFF
Microvirga_lupini                SVYVDKPLIAGLQPDSFASFIGQRSRWAQGMMQILRYKFPPFKRGLKLSQRLCYMSSSMF
Microvirga_lotononidis           SVYVDKPLIAGLQPDSFASFIGQRSRWAQGMMQILRYKFPPLKRGLKISQRLCYMSSSMF
```

```
                                 420       430       440       450       460       470
Rhodobacter_sphaeroides_BcsA     WFFPLVRMMFLVAPLIYLFFGIEIFVATFEEVLAYMPGYLAVSFLVQNALFARQRWPLVS
Citreicella_sp_SE45              WLFPVVRMTFLLSPLFYLFFGLQVFVVQPDEVMAYILPYLLVAFLVQNGLFARVRWPLIS
Salipiger_mucosus_DSM_16094      WLFPVVRMIFLISPLFYLFFGLQIFVVQPDGVMAYILPYLLIAMLVQNGLFARVRWPLIS
Roseivivax_halodurans_JCM_10272  WLFPLIRTIFLVSPLIYLFFGLQIFVVNPAEVLGYILPYLLIGFMVQNALFANVRWPQIS
Roseivivax_atlanticus            WLFPLVRTVFLLSPLLYLFFGLEIFVVTGEEVLAYILPYLLIGFMIQNALFASVRWPQIS
Oceanicola_sp_S124               WLFPLIRMIFLLSPLLYLFFGLEIFVVDSSEVFVYILPYLLIGFIVQNALFSDVRWPQIS
Tistrella_mobilis_KA081020-065   WLFPFSRLMFLIAPLFYLFFGLEIYRATAAEFAGYTLTYLVASLMIQNALNGRFRWPLIS
Methylobacterium_sp_88A          WFFPVPRMIFMFAPMLHIFFDLKIFVASVDESIAYTATYIVINLMMQNYVYGKFRWPFVS
Pelagibacterium_halotolerans     WLFPFTRIIFLIAPFFYLFFNLQIFTASGGEFAAYTMTYLVVNLVMQNSLYGRWRWPWIS
Agrobacterium_tumefaciens        WLFPFPRTIFLFAPLFYLFFDLQIFVASGGEFLAYTAAYMLVNLMMQNFLFGNFRWPWIS
Bosea_sp_117                     WFFPFSRMIFLISPIFYLGFSLEIFNASGAEFLAYTSTYMLVNLLMQNYLYGRYRWPWIS
Microvirga_lupini                WLFPFSRFCFLVSPLCYLFFSLEIFTASGGEFFAYTFTYMMVNFMMQNYLYGRYRWPWIS
Microvirga_lotononidis           WLFPFSRFCFLISPLCYLFFSLEIFTASGGEFLAYTFTYMMVNFMMQNYLYGRYRWPWIS
```

```
                                 480       490       500       510       520       530
Rhodobacter_sphaeroides_BcsA     EVYEVAQAPYLARAIVTTLLRPRSARFAVTAKDETLSENYISPIYRPLLFTFLLCLSGVL
Citreicella_sp_SE45              EVYEIAQTPYLLMAVFKTMLRPHAAKFNVTAKDESLDNDFISPVMKPLFITTLLMFGGLV
Salipiger_mucosus_DSM_16094      EVYEIAQTPYLLKAVFKTFISPRSAKFNVTAKDETLEDEFISPVLVPLLLTTLVLLAGIV
Roseivivax_halodurans_JCM_10272  EVYEIAQTPYLFAALIETVRHPRGASFKVTAKDDTLDRAFMSPIAMPLMVLTGLLLAGIV
Roseivivax_atlanticus            EVYEIAQTPYLLRAVFETVLRPRGATFKVTAKDDTLDSAFLSPIYRPLTLMTGLLLAGLI
Oceanicola_sp_S124               EVYEIAQTPYILLALIETVRNPRGATFKVTAKDGTLEHAFLSPIYRPLTLLVGVLLAGLL
Tistrella_mobilis_KA081020-065   ELYELSQSLHTSVAIVKTMIDPRKPVFRVTAKGETLAHDHVSPLAKPLVGVVGLLILGMG
Methylobacterium_sp_88A          ELYEYVQGLYLFRAIISVIVSPRKPTFNVTAKGAALDQDHLSPMAWPYFAVYFILLIGCG
Pelagibacterium_halotolerans     ELYEYIQSVHLLPAILSVIRNPTKPTFKVTAKDESIDESRLSELARPFYFIFLLLLVGVA
Agrobacterium_tumefaciens        ELYEYVQTVHLLPAVVSVIFNPSKPTFKVTAKDESISEARLSEISRPFFVIFGVLVIAMI
Bosea_sp_117                     ELYEYIQSVYLLPALISVILNPRKPTFKVTAKGETLSESHVSEIGKPFFIIFALQIVAAL
Microvirga_lupini                DLYEYIQTIYLLPAVVSVIANPSKPTFKVTAKNESMEESRVSELGTPYFIIFGILILGVI
Microvirga_lotononidis           DLYEYIQTIYLLPAVLSVIANPSKPTFKVTAKNESMEESRVSELGTPYFIIFGILILGVI
```

```
                                 540       550       560       570       580       590
Rhodobacter_sphaeroides_BcsA     ATLVRWVAFPGDRSVLLVVGGWAVLNVLLVGFALRAVAEKQQRRAAPRVQMEVPAEAQIP
Citreicella_sp_SE45              AAGIRWYMYPGDRLIVQIVGGWALFNFILTVLSLRSVFERQRRLRKPRTEIMVPAAVWSE
Salipiger_mucosus_DSM_16094      AAALRWYLFPGDRSVVQIVGGWALFNLLLAILALRSVIERQRRLLKPRTAIMAPASLWPE
Roseivivax_halodurans_JCM_10272  AAVARYIAFPGDRTVVQIVAAWNVYNFLLAALALRAVFERPARLVRPRTAVFVPARLHIP
Roseivivax_atlanticus            AGLIRWIAFPGDRGVIQIVGAWNLYNFLLAAFALRAVFERPWRLVKPRTAVSTPARLALS
Oceanicola_sp_S124               AGVYRWYAFPGDRYVVQIVGAWALYNFILAAFSLRAVFERPRRLTRPRTAVSTPARLTLV
Tistrella_mobilis_KA081020-065   AALWRWWMEPATRDVLLVVTFWNTFNLLLASAALGVVCEHAQHRAAPRVMVKRPARILID
Methylobacterium_sp_88A          TALYRYLFEPGVTNLMLVVGLWNFFNLLTAGVALGVAAERRTTEASPSLAIDRQGRLVFG
Pelagibacterium_halotolerans     FTGYRLWAEPYRAEITLVVGGWNLFNLILAGCALGVVSERREVRASRRVPIERRCEIRSV
Agrobacterium_tumefaciens        FAIYRVYAEPYKADVTLVVGGWNLLNIIFAGCALGVVSERGEKSASRRVTVKRRCELQIG
Bosea_sp_117                     ITIWRIMTEPYTADVTLVVGGWNLLNLMISGCALGVVSERANRRRTHRVNLSRRCQLIVN
Microvirga_lupini                ATGVRVWAEPYKADLTLVTGAWNVLNLIIAGCALGVVSERATRRQSHRVRVERPCRFIMG
Microvirga_lotononidis           ATGIRVWAEPYKADLTLVTGAWNVLNLIIAGCALGVVSERATRRHSHRVRVERPCRFIIG
```

```
                                    600         610         620         630         640
Rhodobacter_sphaeroides_BcsA        AFGNRSL........TATVLDASTSGVRLLVRL....PGVGDPHPALEAGGLIQ...FQP
Citreicella_sp_SE45                 SKTDGAGVS.......AVLRMSSGRTMQFTLNDDDE..ARE.ELGMIRSGDRLV...LCP
Salipiger_mucosus_DSM_16094         GQHSGSATD.......VVLRAISARKMRFVFADDDR..KNE....MIGEGARLV...IRP
Roseivivax_halodurans_JCM_10272     GADDDAA......DLETTIISASPTVIQARLAAGALPKGLAPG.SEGAAPMRGK...LSP
Roseivivax_atlanticus               GDETGAGA....TMLDVTIIAATAGSVQLRLDPDSGDSATRDWKKLSIKGALAT...LTP
Oceanicola_sp_S124                  PAESDQSPAPSGEPREATILSASPERIQLQLT....ESGQESWTPEQ.VGAEVV...LLP
Tistrella_mobilis_KA081020-065      G...........TLYEGALLDLSQGGARLAIPARQAPKGRLAG.....RFAELLVDELTA
Methylobacterium_sp_88A             G...........RALDVAIERASALGCTIRFGAEGLPP...GA......GSTHRMGQLSV
Pelagibacterium_halotolerans        D.G.........TWVRGTILNVSSGGVAIQVADG.KTRLAKDQ......ATAIRFQTLSP
Agrobacterium_tumefaciens           DND.........AWMPATIENVSVNGMLIHIFEEGLSVVEKGS......KAVIRVKPHSE
Bosea_sp_117                        D...........VAYPAIIEDVSIGGAGIRTAAGNLPKIDRGQ......IASIRFKPQSD
Microvirga_lupini                   D...........EIIDAVLKDVSVGGARVHVPPSAEPKLKKGA......SGTLEFQPFSS
Microvirga_lotononidis              D...........EAIDAVVRDVSVGGARVHVPPSAEPKLKKGA......AGTLEFQPFSN

                                            650         660         670         680         690
Rhodobacter_sphaeroides_BcsA        KFPDAPQLERMVRGR...IRSARREGGTVMVGVIFEAGQPIAVRETVAYLIFGESAHWRT
Citreicella_sp_SE45                 VIPAAPDLEHHLRLHVTRVSHDEQAGRGTAVTVEVEDGQLIPASEITAYLVYGNSEKWVA
Salipiger_mucosus_DSM_16094         KIDDAPELQHELTITLTRAEDPSDNAGRRLWSADIDRDHLIAASEITAHLVYGDSEKWMA
Roseivivax_halodurans_JCM_10272     VLPKSPALQTPLNVTVNEVRSDP...DGLLLELGISAEQMVDASRLTAVIVYGDSSRWEF
Roseivivax_atlanticus               ILEKTPQLEAPLEVSISDVSSGA...DGIRVGLVVDPAHMIAASHLSATVVYGDSSRWHF
Oceanicola_sp_S124                  ILPKAADLEVPLTLTLAGVRPGV...EGTRVDLAVPADSMIGAARLTAHVVYGDSRRWQF
Tistrella_mobilis_KA081020-065      PAHLATGEARPVPVEMRYG..RPDDAGNIQIGLRFMPETDAA.KEAIVDLVYGSSALWAE
Methylobacterium_sp_88A             VPIDGAPVQVPLPVEIGEI...TQSGDEVVVTLQFQPLDPKG.YGALASLMYGDAGALLR
Pelagibacterium_halotolerans        VPDN......EMGIFVRNV...SNAGKSSMVGCRFMAEKPSD.YRLIADLLYANSKLWKE
Agrobacterium_tumefaciens           GVPE......TMPINIVRS...VRQNGYTSVGSTFSPQQAVD.HRLIADLIFANSDQWSE
Bosea_sp_117                        IGID......TLPLAIRNV...KREGAEHLLGCEFHPEIPLH.HRLIADLIFANADEWQK
Microvirga_lupini                   LPVQ......HLPMEIRKV...GMDDRGLLLGCRFMIEKPEH.RRMIADLVFANADQWSE
Microvirga_lotononidis              LPIQ......HLPMEIRKV...GMDDKGLLLGCRFLIEKPEH.RRMIADLVFANADQWSQ

                                   700         710         720         730         740         750
Rhodobacter_sphaeroides_BcsA        MREATMRPIGLLHGMARILWMAAASLPKTARDFMDEPARRRRRHEEPKEKQAHLLAFGT.
Citreicella_sp_SE45                 AREALPQARGLISGIVFVLRLSVGSIVPSVLALVTHRAAPQHNRKDEDRN.......TVE
Salipiger_mucosus_DSM_16094         AREAVPRSRGLISGIVFVLRLSLTSIVPSAIALFTYRGAPQDNPEDRYRN.......ANE
Roseivivax_halodurans_JCM_10272     FRKVVPRSSTLVAGLAYVLGKAALSLPKTTLDLLREPGRRQSEMLEEDAVRENTFSAAPE
Roseivivax_atlanticus               FRKVRPQSSTLLAGLVYVLAKSVVSIPKSVYDFLREPARRRDQLAADLTLPVDSFAEASK
Oceanicola_sp_S124                  FRKFHAQGSTLVTGLLYVLAKSALSVPQTIYDFLREFSK.....................
Tistrella_mobilis_KA081020-065      LLSRRHRRPGVVRGVAHFIRLSAAYGFRSLGFVLGG.SRFRANPAPPPPP..RLQ.....
Methylobacterium_sp_88A             FQQSRRRHKNILSGTLQLIWWGLVEPFRAASYAFKA.GHASTEEAATKAA..KAPAVLPK
Pelagibacterium_halotolerans        RQESRQVNIGIILGTLQFLSISIYQTARGLGYLARF.SRPAGSDQPQEAR..S.......
Agrobacterium_tumefaciens           LQSSRRKNPGLIKGTATFLAIAVFQTNRGLFYLFRS.FVPEKKPKPTVER..VKT.....
Bosea_sp_117                        FQYSRRHNPGVLKGTLQFIGIAIYQTARGLSYMMQL.HRLQRAASRSTVS..TNK.....
Microvirga_lupini                   FQKNRHHDIGVLRGTLWFFMVAFYQTGRGLSYFFGL.QKIGTSNPPAPSV..ASA.....
Microvirga_lotononidis              FQKNRHHDIGVLRGTLWFFMVAFYQTGRGLSYFFGL.QKIGASKPSAPSV..ASA.....

                                                                760         770         780         790
Rhodobacter_sphaeroides_BcsA        .........DF............STEPDWAGELLDPTAQVSARPNTVAWGSNHHHHHHKL
Citreicella_sp_SE45                 KTAIEHYLADR............GALPAPATEFLVPEETG.GAP................
Salipiger_mucosus_DSM_16094         TEAIKYYLADN............TSDSDLSAAFLLPEDEG.ART................
Roseivivax_halodurans_JCM_10272     KARTKPA................GRADLDDDGYLTVEE......................
Roseivivax_atlanticus               EQ.EKPAEPVR............QRRRSAESQYLEIED......................
Oceanicola_sp_S124                  ............................................................
Tistrella_mobilis_KA081020-065      ...TEPASAQ....EGVPPDGNLTPPTGWSGS........ATAPRTS.............
Methylobacterium_sp_88A             PEAKAPLLVQRPAPFTAPGDTPPTPPPGPSDNPLVVRQGSSRIPGSEAATDWVRL...ML
Pelagibacterium_halotolerans        ............................................................
Agrobacterium_tumefaciens           ............................................................
Bosea_sp_117                        ..A.........................................................
Microvirga_lupini                   ..VK........................................................
Microvirga_lotononidis              ..AK........................................................

                                    800
Rhodobacter_sphaeroides_BcsA        HHHHHH....................
Citreicella_sp_SE45                 ..........................
Salipiger_mucosus_DSM_16094         ..........................
Roseivivax_halodurans_JCM_10272     ..........................
Roseivivax_atlanticus               ..........................
Oceanicola_sp_S124                  ..........................
Tistrella_mobilis_KA081020-065      ..........................
Methylobacterium_sp_88A             EFENDRLLEGAGKQANRGSSDPLVRT
Pelagibacterium_halotolerans        ..........................
Agrobacterium_tumefaciens           ..........................
Bosea_sp_117                        ..........................
Microvirga_lupini                   ..........................
Microvirga_lotononidis              ..........................
```

REFERENCES

(1) Brooks, B. R.; Brooks, C. L.; Mackerell, A. D.; Nilsson, L.; Petrella, R. J.; Roux, B.; Won, Y.; Archontis, G.; Bartels, C.; Boresch, S.; Caflisch, A.; Caves, L.; Cui, Q.; Dinner, A. R.; Feig, M.; Fischer, S.; Gao, J.; Hodoscek, M.; Im, W.; Kuczera, K.; Lazaridis, T.; Ma, J.; Ovchinnikov, V.; Paci, E.; Pastor, R. W.; Post, C. B.; Pu, J. Z.; Schaefer, M.; Tidor, B.; Venable, R. M.; Woodcock, H. L.; Wu, X.; Yang, W.; York, D. M.; Karplus, M. *J. Comput. Chem.* **2009**, *30*, 1545.

(2) Jo, S.; Kim, T.; Iyer, V. G.; Im, W. *J. Comput. Chem.* **2008**, *29*, 1859.

(3) Jo, S.; Kim, T.; Im, W. *PLoS One* **2007**, *2*.

(4) Jo, S.; Lim, J. B.; Klauda, J. B.; Im, W. *Biophys. J.* **2009**, *97*, 50.

(5) Wu, E. L.; Cheng, X.; Jo, S.; Rui, H.; Song, K. C.; Davila-Contreras, E. M.; Qi, Y. F.; Lee, J. M.; Monje-Galvan, V.; Venable, R. M.; Klauda, J. B.; Im, W. *J. Comput. Chem.* **2014**, *35*, 1997.

(6) Gorchein, A. *P. Roy. Soc. B* **1968**, *170*, 270.

(7) Benning, C.; Huang, Z. H.; Gage, D. A. *Arch. Biochem. Biophys.* **1995**, *317*, 103.

(8) Hsin, J.; Gumbart, J.; Trabuco, L. G.; Villa, E.; Qian, P.; Hunter, C. N.; Schulten, K. *Biophys. J.* **2009**, *97*, 321.

(9) Best, R. B.; Zhu, X.; Shim, J.; Lopes, P. E. M.; Mittal, J.; Feig, M.; MacKerell, A. D. *J. Chem. Theory Comput.* **2012**, *8*, 3257.

(10) MacKerell, A. D.; Bashford, D.; Bellott, M.; Dunbrack, R. L.; Evanseck, J. D.; Field, M. J.; Fischer, S.; Gao, J.; Guo, H.; Ha, S.; Joseph-McCarthy, D.; Kuchnir, L.; Kuczera, K.; Lau, F. T. K.; Mattos, C.; Michnick, S.; Ngo, T.; Nguyen, D. T.; Prodhom, B.; Reiher, W. E.; Roux, B.; Schlenkrich, M.; Smith, J. C.; Stote, R.; Straub, J.; Watanabe, M.; Wiorkiewicz-Kuczera, J.; Yin, D.; Karplus, M. *J. Phys. Chem. B* **1998**, *102*, 3586.

(11) Mackerell, A. D.; Feig, M.; Brooks, C. L. *J. Comput. Chem.* **2004**, *25*, 1400.

(12) Hart, K.; Foloppe, N.; Baker, C. M.; Denning, E. J.; Nilsson, L.; MacKerell, A. D. *J. Chem. Theory Comput.* **2012**, *8*, 348.

(13) Guvench, O.; Greene, S. N.; Kamath, G.; Brady, J. W.; Venable, R. M.; Pastor, R. W.; Mackerell, A. D. *J. Comput. Chem.* **2008**, *29*, 2543.

(14) Guvench, O.; Hatcher, E.; Venable, R. M.; Pastor, R. W.; MacKerell, A. D. *J. Chem. Theory Comput.* **2009**, *5*, 2353.

(15) Guvench, O.; Mallajosyula, S. S.; Raman, E. P.; Hatcher, E.; Vanommeslaeghe, K.; Foster, T. J.; Jamison, F. W.; MacKerell, A. D. *J. Chem. Theory Comput.* **2011**, *7*, 3162.

(16) Durell, S. R.; Brooks, B. R.; Bennaim, A. *J Phys Chem-Us* **1994**, *98*, 2198.

(17) Jorgensen, W. L.; Chandrasekhar, J.; Madura, J. D.; Impey, R. W.; Klein, M. L. *J. Chem. Phys.* **1983**, *79*, 926.

(18) Phillips, J. C.; Braun, R.; Wang, W.; Gumbart, J.; Tajkhorshid, E.; Villa, E.; Chipot, C.; Skeel, R. D.; Kale, L.; Schulten, K. *J. Comput. Chem.* **2005**, *26*, 1781.

(19) Miyamoto, S.; Kollman, P. A. *J. Comput. Chem.* **1992**, *13*, 952.

(20) Ryckaert, J. P.; Ciccotti, G.; Berendsen, H. J. C. *J Comput Phys* **1977**, *23*, 327.

(21) Darden, T.; York, D.; Pedersen, L. *J. Chem. Phys.* **1993**, *98*, 10089.

(22) Nam, K.; Gao, J. L.; York, D. M. *J. Chem. Theory Comput.* **2005**, *1*, 2.

(23) Walker, R. C.; Crowley, M. F.; Case, D. A. *J. Comput. Chem.* **2008**, *29*, 1019.

(24) Crowley, M. F.; Williamson, M. J.; Walker, R. C. *Int. J. Quantum Chem.* **2009**, *109*, 3767.

(25) Case, D. A.; T.A. Darden; T.E. Cheatham, I.; C.L. Simmerling; J. Wang; R.E. Duke; R. Luo; R.C. Walker; W. Zhang; K.M. Merz; B. Roberts; S. Hayik; A. Roitberg; G. Seabra; J. Swails; A.W. Goetz; I. Kolossváry; K.F. Wong; F. Paesani; J. Vanicek; R.M. Wolf; J. Liu; X. Wu; S.R. Brozell; T. Steinbrecher; H. Gohlke; Q. Cai; X. Ye; J. Wang; M.-J. Hsieh; G. Cui; D.R. Roe; D.H. Mathews; M.G. Seetin; R. Salomon-Ferrer; C. Sagui; V. Babin; T. Luchko; S. Gusarov; A. Kovalenko; Kollman, P. A. University of California, San Francisco, 2012.

(26) Morgan, J. L. W.; McNamara, J. T.; Zimmer, J. *Nat. Struct. Mol. Biol.* **2014**, *21*, 489.

(27) Morgan, J. L. W.; Strumillo, J.; Zimmer, J. *Nature* **2013**, *493*, 181.

(28) Kumar, S.; Bouzida, D.; Swendsen, R. H.; Kollman, P. A.; Rosenberg, J. M. *J. Comput. Chem.* **1992**, *13*, 1011.

(29) Grossfield, A.; 2.0.6 ed.; Grossfield, A., Ed. 2012.

(30) French, A. D.; Johnson, G. P. *Cellulose* **2004**, *11*, 449.

(31) French, A. D.; Johnson, G. P.; Cramer, C. J.; Csonka, G. I. *Carbohydr. Res.* **2012**, *350*, 68.

(32) French, A. D.; Johnson, G. P.; Kelterer, A.-M.; Dowd, M. K.; Cramer, C. J. *International Journal of Quantum Chemistry* **2001**, *84*, 416.

(33) Johnson, G. P.; Petersen, L.; French, A. D.; Reilly, P. J. *Carbohydr. Res.* **2009**, *344*, 2157.

(34) Gouet, P.; Robert, X.; Courcelle, E. *Nucleic Acids Res.* **2003**, *31*, 3320.
